# PKD1 gene mutation and ultrasonographic characterization in cats with renal cysts

**DOI:** 10.12688/f1000research.134906.1

**Published:** 2023-06-28

**Authors:** Kotchapol Jaturanratsamee, Palin Jiwaganont, Pratch Sukumolanan, Soontaree Petchdee

**Affiliations:** 1 Graduate School, Bio-Veterinary Science Program, Faculty of Veterinary Medicine, Kasetsart University, Bangkok, Bangkok, Thailand; 2 Graduate School, Veterinary Clinical Studies Program, Faculty of Veterinary Medicine, Kasetsart University, Bangkok, Bangkok, Thailand; 3 Department of Large Animal and Wildlife Clinical Sciences, Faculty of Veterinary Medicine, Kasetsart University, Bangkok, Bangkok, Thailand

**Keywords:** Gene, kidney disease, mutation, polycystic, ultrasonography

## Abstract

**Background:** Polycystic kidney disease (PKD) has a complex phenotype partly explained by genetic variants related to this disease. Ultrasonography is a promising approach for defining clinical signs. This study aimed to assess kidney characteristics in cats with Polycystin-1 (PKD1) gene mutations and wild-type cats. Kidney characteristics were identified by ultrasonography.

**Methods:** A total of 108 cats of variable breeds aged an average of 37.01±3.50 months were included. Blood examination and biochemical tests were evaluated. For cystic formation, renal ultrasound was performed. The PKD1 gene mutation was identified *via* polymerase chain reaction (PCR) and DNA sequencing. Matrix correlation and effectiveness of ultrasound for PKD1 mutation detection were determined.

**Results:** The results showed that 19.44% of cats had PKD1 mutations, a high prevalence in Persian and Persian-related breed cats. Our results demonstrated the characteristics of kidneys in wild-type cats and cats with gene mutations. Based on ultrasonography results, there was an association between cats with gene mutations and cyst formation. The findings indicated that ultrasound did not detect cysts in cats aged 4-36 months, supporting the evidence that PKD1 gene mutations may not be present. This study found high sensitivity and renal specificity ultrasound for PKD1 heterozygous mutation. Moreover, cystic formation *via* renal ultrasound showed an increased risk for PKD1 mutation 2,623 times compared to normal kidneys.

**Conclusions:** Ultrasonographic examination, coupled with genetic investigations, may help to clarify the phenotypic variability of PKD1. The phenotypic profile of PKD1 will guide therapeutic outcomes and reduce the prevalence of PKD morbidity and mortality in cats.

## Introduction

The most common hereditary disease in cats is feline polycystic kidney disease (PKD). A previous study reported that the genetic condition of the PKD gene affects Persian and Persian-related cats. ^1^ Renal, hepatic, and pancreatic cysts are the common symptoms of feline PKD, inherited in an autosomal dominant manner. ^2^ This disease is frequently detected in the Persian breed. It is one of this breed’s most common feline genetic diseases, other than diabetes and feline lower urinary tract disease. ^3^ This disease not only impacts the Persian breed but other breeds, including the Exotic Shorthair, British Shorthair, American Shorthair, Himalayan, Scottish fold, Ragdoll, Chartreaux, and Maine Coon breeds, may also be affected by this disease. ^4^^–^^6^ The feline Polycystin-1 (PKD1) gene was examined for the point of 85% of causal mutations, and the cats carried heterozygous CA transversion at c.10063 in exon 29, resulting in impaired renal function. ^1^^,^^7^

Polycystin-1 upregulation was reported in the renal tissue from a mouse model with a PKD1 mutation. The increased expression of the mammalian target of rapamycin (mTOR) affected autophagy and apoptosis signaling in cells with a PKD1 gene mutation. ^1^^,^^7^^–^^9^ In addition, many studies have shown the mechanisms by which cyclic adenosine monophosphate (cAMP) levels influence PKD. ^10^^–^^13^ Oral administration of antioxidant agents such as DHA-enriched fish oils showed potential renoprotective effects in cats with chronic kidney disease related to PKD gene mutation. ^14^

Ultrasonography is a useful diagnostic tool for evaluating the renal structure and renal parenchyma that cannot be evaluated through radiography. ^15^^,^^16^ This technique is a useful diagnostic tool for assessing and revealing renal parenchyma alteration, as that occurs when the disease progresses. ^17^ This technique is a practical and accurate approach to diagnosing PKD. Cats with PKD have multiple hypoechoic to anechoic cysts that are round or oval in shape and clearly distinguished from the renal parenchyma. ^18^ In a previous study, ultrasound had a sensitivity of 75% when performed at 16 weeks of age and 91% when performed at 36 weeks of age. ^19^

This study aims to investigate the association between clinical presentations and the ultrasonography of the kidneys in wild-type cats and cats with gene mutations to provide the guide treatment and aid prevalence estimates of PKD in cats.

## Methods

### Animals

**Figure 1.**
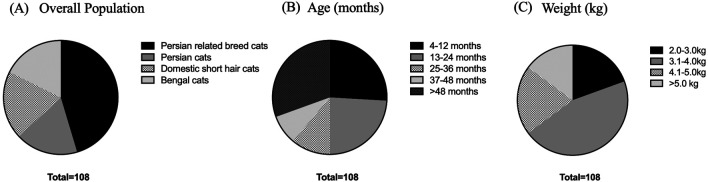
Overall population in this study. (A) Various breeds of enrolled cats. (B) Age in the different groups, including 4-12 months, 13-24 months, 25-36 months, 37-48 months, and more than 48 months. (C) Weight comprising 2.0-3.0, 3.1-4.0, 4.1-5.0, and more than 5.0 kg.

The Ethics Committee of Kasetsart University, Bangkok, Thailand, approved this research on September 12, 2022 (ACKU-65-VET-077). In addition, all cat owners in this study signed an informed consent agreement. ^38^ A total of 108 cats, including 18 Persian cats, 49 Persian-related breed cats, 22 domestic shorthair cats, and 19 Bengal cats, were enrolled in this study at the Veterinary Teaching Hospital, Faculty of Veterinary Medicine, Kasetsart University, from September 2022 to April 2023, as shown in Figure 1. The cat underwent a complete physical examination to evaluate the general condition. All procedures were refined as much as possible to minimize suffering to alleviate the harm to the cats in this study with a high standard that is internationally accepted (‘Best Practice Guidelines’) of veterinary clinical care for individual cats and following the ethical approvals. This study adhered to the ARRIVE guidelines. ^39^

**Figure 2.**
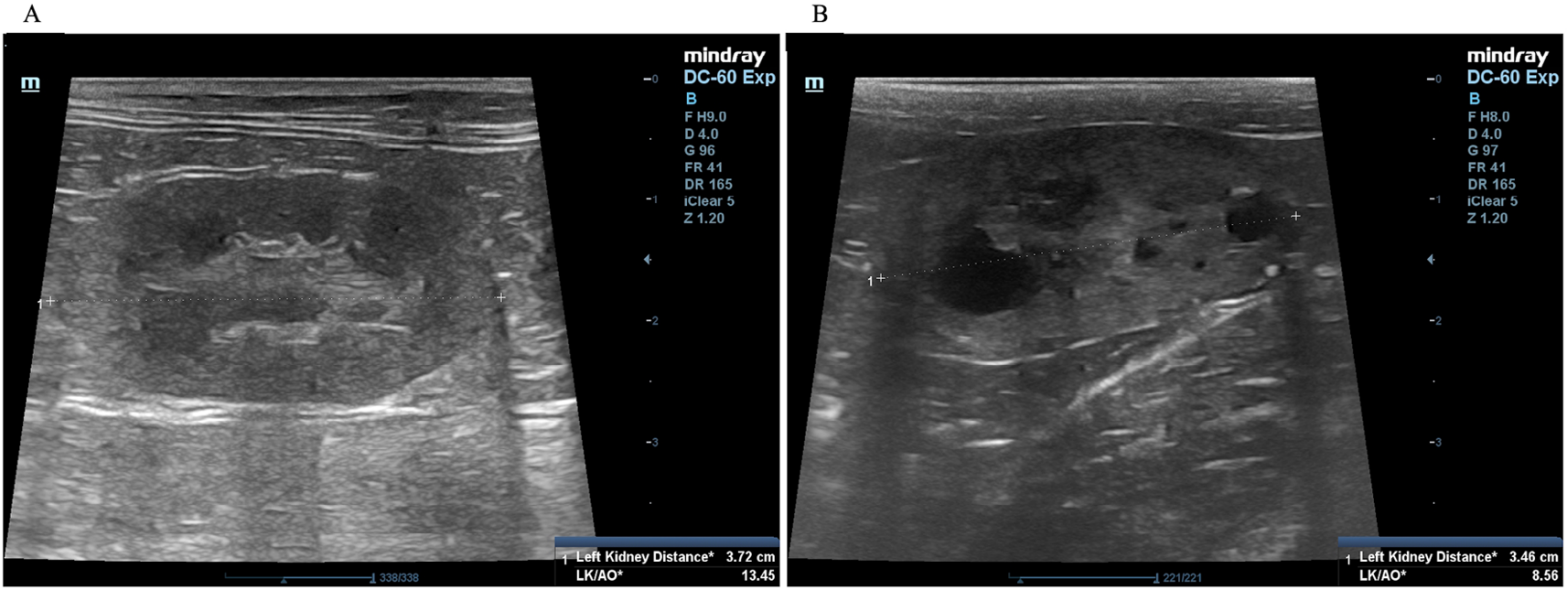
Ultrasound image of kidneys with cysts. (A) Normal kidney appearance without cystic formation. (B) Multiple cyst formation in renal parenchyma. In (A) and (B), the ultrasonography image was cropped for the proper size.

A complete blood count and serum biochemistry profile were performed for each cat. Kidney characterization was assessed by ultrasonography analysis in all cats using only gentle restraint and no sedation. The non-invasive two-dimensional (2D)-mode ultrasonography was used to define the kidney structure and size. The ultrasonography images have demonstrated that multiple cysts are a common finding in PKD1 heterozygous mutation cats, as shown in Figure 2.

### Polymerase chain reaction and DNA sequencing

DNA amplification with polymerase chain reaction (PCR) was performed to detect PKD1 gene mutation. PCR protocol obtained the specific forward and reversed primers from Lee *et al*. ^18^ The PCR primers were PKD-forward 5′-CAGGTAGAC GGGATAGACGA-3′ and PKD-reverse 5′-TTCTTCCTGGTCAACGACTG-3′. In brief, DNA was extracted from the blood using FavorPrep Blood Genomic DNA Extraction Mini Kit (Farvogen, Taiwan). Then, 2 μl of extracted DNA from blood (Farvogen, Taiwan) was mixed with 2.5 μl of 10X Taq buffer, 0.2 μl of dNTPs (25 mM each dNTP) (Thermo Fisher Scientific, USA), 1.5 μl of MgCl _2_ (25 mM), 1 μl of each custom forward and reverse primer from the manufacturer (Integrated DNA Technologies, USA) (10 μM each primer), 1 unit of Taq DNA polymerase (Thermo Fisher Scientific, USA), and 16.7 μl of nuclease-free water. The final volume of the PCR reaction was 25 μl per reaction. The PCR condition was as follows: heat-activation at 94°C for 3 minutes, 35 cycles of denaturation for 1 minute, annealing at 58°C for 1 minute, and extension at 72°C for 1 minute. Finally, the final extension was performed at 72°C for 10 minutes *via* Biometra Thermocycler T-Gradient ThermoBlock (Thermo Fisher Scientific, USA). The PCR product was detected with 1.5% agarose gel electrophoresis. The PCR product was visualized under gel documentation (Bio-rad, USA) at 559 bp. The PCR product was stored at -20°C until sequencing. The Barcode-tagged (BT) sequencing method was performed to detect the PCR product’s nucleotide. According to BT sequencing, the PCR product was analyzed by Celemics, Inc. (Seoul, Korea). Single nucleotide polymorphism of the PKD1 gene was detected using the BioEdit version 7.2 (RRID:SCR_007361) program and A plasmid Editor (ApE) version 2.0.60 (RRID:SCR_014266) program.

### Ultrasonography

The procedure for ultrasound examination of the kidneys followed that of a previous report. ^4^^,^^20^ Briefly, ultrasonography was performed using a Mindray real-time ultrasound machine (model DC-7, Shenzhen Mindray Bio-medical Electronics, Nanshan, Shenzhen, China) with a linear transducer (frequency 7.5-12.0 MHz). The kidneys were examined in the longitudinal and transverse planes. Results were recorded for each cat. The three separated regions, the renal cortex, the renal medulla, and the renal sinus, were identified. The renal sinus is the most hyperechoic component of the kidney and is surrounded by the renal pelvis and vascular branches. In the renal pelvis, ultrasonography images can be assessed using the renal crest as a landmark. The renal size can be measured by measuring from the long axis view. The renal lengths of the right and left kidneys were measured to identify the renal structure, as shown in Figure 2.

### Statistical analysis

Descriptive statistics were determined as mean ± standard error of the mean (SEM). An evaluation of the effectiveness of diagnostic tests was performed. The datasets were analyzed using a paired Student’s- t-test. An odds ratio (OD) was performed to measure the association between cysts in renal parenchyma and PKD1 mutation. A correlation matrix was used to represent the correlation coefficients for different variables. Pearson’s correlation coefficient was used to determine the correlation between variables and to analyze the multiple linear regression models that contained several independent variables (GraphPad Prism (RRID: SCR_002798) Software version 9.0, USA). A *P*-value of 0.05 or less was indicated for statistical significance.

## Results

### Characteristics, ultrasonographic variables, and serum biochemical findings

The number of cats in the PKD1 mutation group was 21 (19.44 %) from an overall population of 108 cats. The cats in the PKD1 mutation group were older than those in the wild-type group (50.89 ± 9.19 *vs.* 33.93 ± 3.59 months, *P* = 0.051). However, there were no significant differences in weight between cats in the wild-type group and the PKD1 mutation group. General characteristics and ultrasonographic parameters for the cats are reported in Table 1. ^36^^,^^37^ The size of the left kidney was significantly larger in the PKD1 mutation group than in the wild-type group. Blood profiles and serum biochemistry results are reported in Table 2. The levels of other biochemical profiles, such as symmetric dimethyl arginine creatinine (SDMA), significantly differed between groups. However, the blood profile parameters of red blood cell count, hematocrit, and plasma protein were not different between the groups.

**Table 1.** Characteristics and ultrasonographic variables of cats.

Parameters (Mean ± SEM)	PKD1		*P* value
	Wild-type group	HET mutant group	
Age in months	33.93 ± 3.59	50.89 ± 9.19	*P* = 0.051
Weight (kg)	4.36 ± 0.46	3.34 ± 0.22	*P* = 0.283
Male (number [%])	72.4	61.9	-
Right kidney size (cm)	3.64 ± 0.39	4.44 ± 0.22	*P* = 0.082
Left kidney size (cm)	3.54 ± 0.37 *	4.58 ± 0.28 *	*P* = 0.036
Cysts number	0	12.61 ± 2.09	-

PKD1 = Polycystin-1, HET = heterozygous mutation.

Values are presented as the mean ± SEM.

* *P* < 0.05.

**Table 2.** Serum biochemistry, red blood cell count, and hematocrit in all cats and groups.

Parameters (Mean ± SEM)	Overall population	Wild-type group	PKD1 mutant group	Reference value
BUN (mg%)	42.81 ± 3.54	38.18 ± 4.05	50.35 ± 11.39	15-34
Creatinine (mg%)	2.21 ± 0.19	1.95 ± 0.21	2.00 ± 0.33	<2.0
BUN/Creatinine ratio	19.4	19.6	25.2	7-37
Hematocrit (%)	35.87 ± 0.63	37.30 ± 3.95	33.01 ± 1.35	30-45
PP (g/dL)	7.64 ± 0.08	7.54 ± 0.80	8.0 ± 0.19	5-7.5
SDMA (ug/dL)	12.33 ± 0.74	9.36 ± 0.999.36 ***	20.5 ± 2.06 ***	<14

PKD1 = Polycystin-1, BUN = blood urea nitrogen, PP = plasma protein, SDMA = symmetric dimethylarginine.

*** *P* < 0.001.

### Sequencing results and ultrasonography correlation analysis of PKD1 in cats

**Figure 3.**
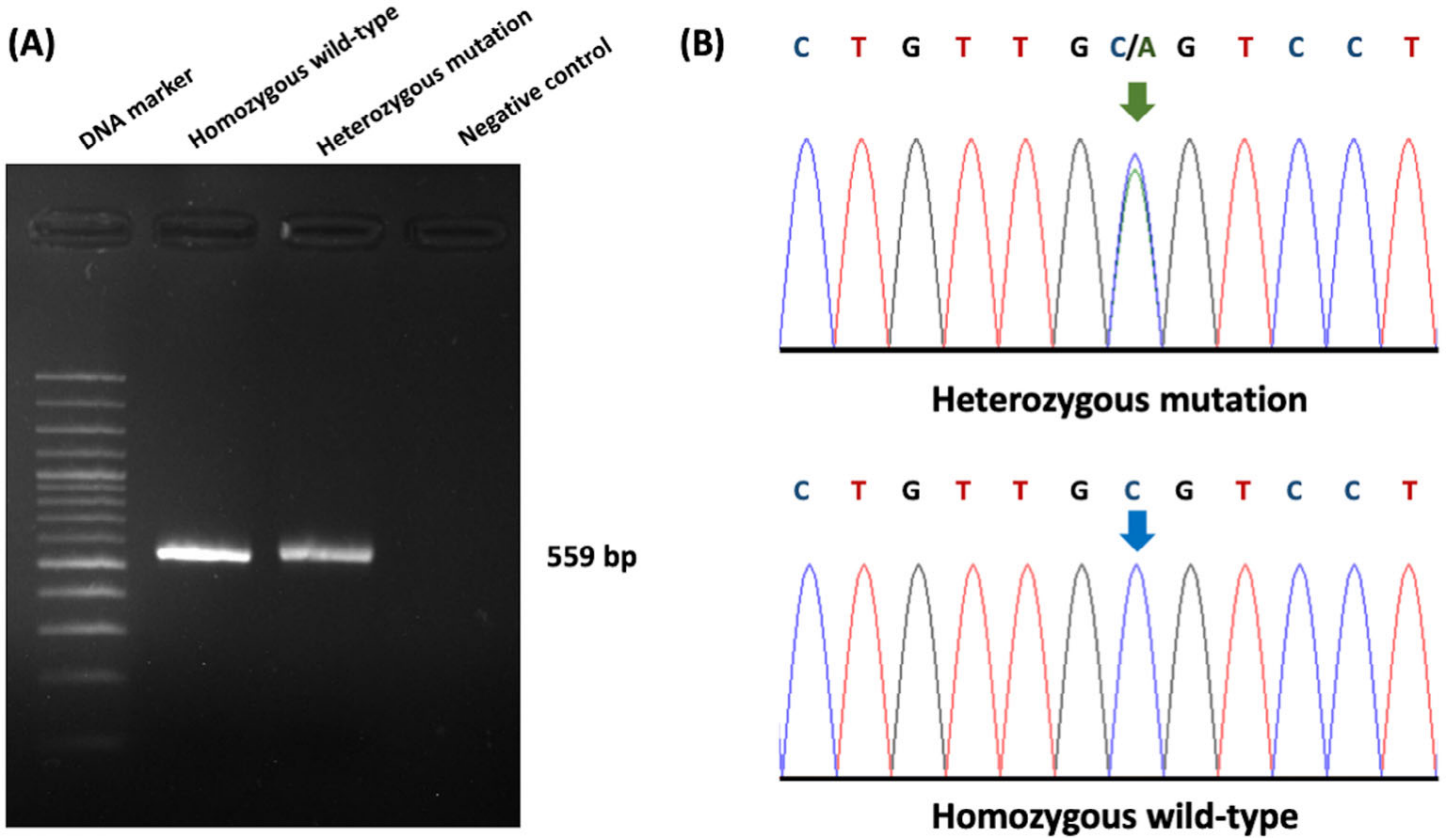
Results of Polycystin-1 (PKD1) polymorphism from genotyping by sequencing. (A) Gel electrophoresis represented 559 bp of target DNA of the PKD1 gene. Lane 1 represents DNA marker (M), while Lane 2 and Lane 3 show DNA products from cats with homozygous wild-type and heterozygous mutation, respectively. Lane 4 indicates negative control. (B) The barcode-tagged (BT) sequencing result of PKD1 was shown in two groups, including homozygous wildtype (C/C) and heterozygous mutation (C/A). The green arrow pointed to two peaks of cytosine and alanine, while the blue arrow represented one peak of cytosine. In (A), the gel electrophoresis image was adjusted to the darkness of the background and cropped for the proper size. In (B), the chromatogram results were cropped from BT sequencing in the Bioedit program and the arrow cursors (green and blue) were added, pointing to the mutation area for clarity and easy understanding. The text of the chromatogram nucleotide above the sequencing picture was edited in another font with bold letters.

The association between ultrasonography findings and the genotypes of PKD1 mutations was analyzed for 108 cats. PKD1 heterozygous mutations were identified in 21 cats, and the results showed significant differences between C/C and C/A genotypes for the homozygous wild-type and heterozygous mutation ( Figure 3). In the present study, gene mutation cats were associated with cyst formation in cats. The ultrasonographic analysis showed renal cysts in 19 cats, and all of these cats harbored PKD1 heterozygous mutations.

### Effectiveness of ultrasonography for detecting PKD1 gene mutation

Regarding renal ultrasonography, the effectiveness of this method was evaluated for detecting the variation of PKD1 gene polymorphism ( Table 3). The sensitivity and specificity of renal ultrasound accounted for 100.00% (95% CI, 84.54 to 100.00) and 98.91% (95% CI, 94.10 to 99.94), respectively. In addition, positive predictive value (PPV) and negative predictive value (NPV) were indicated at 95.45% (95% CI, 78.20 to 99.77) and 100.00% (95% CI, 95.95 to 100.00), respectively. On top of that, according to the OD, presenting renal cysts from renal ultrasonography increased the possibility for PKD1 heterozygous mutation 2,623.00 times (95% CI, 103.2465 to 66637.8792, *P* < 0.0001).

**Table 3.** Results of the effectiveness of ultrasonography for detecting PKD1 heterozygous mutation.

Statistical test	Value	95% Confidence Interval
Sensitivity	100.00%	84.54% to 100.00%
Specificity	98.91%	94.10% to 99.94%
Positive Predictive Value (PPV)	95.45%	78.20% to 99.77%
Negative Predictive Value (NPV)	100.00%	95.95% to 100.00%
Odds ratio (OD)	2623.00	103.2465 to 66637.8792

PKD1 = Polycystin-1.

### Relationships between renal cysts and PKD1 genotypes in cats

**Figure 4.**
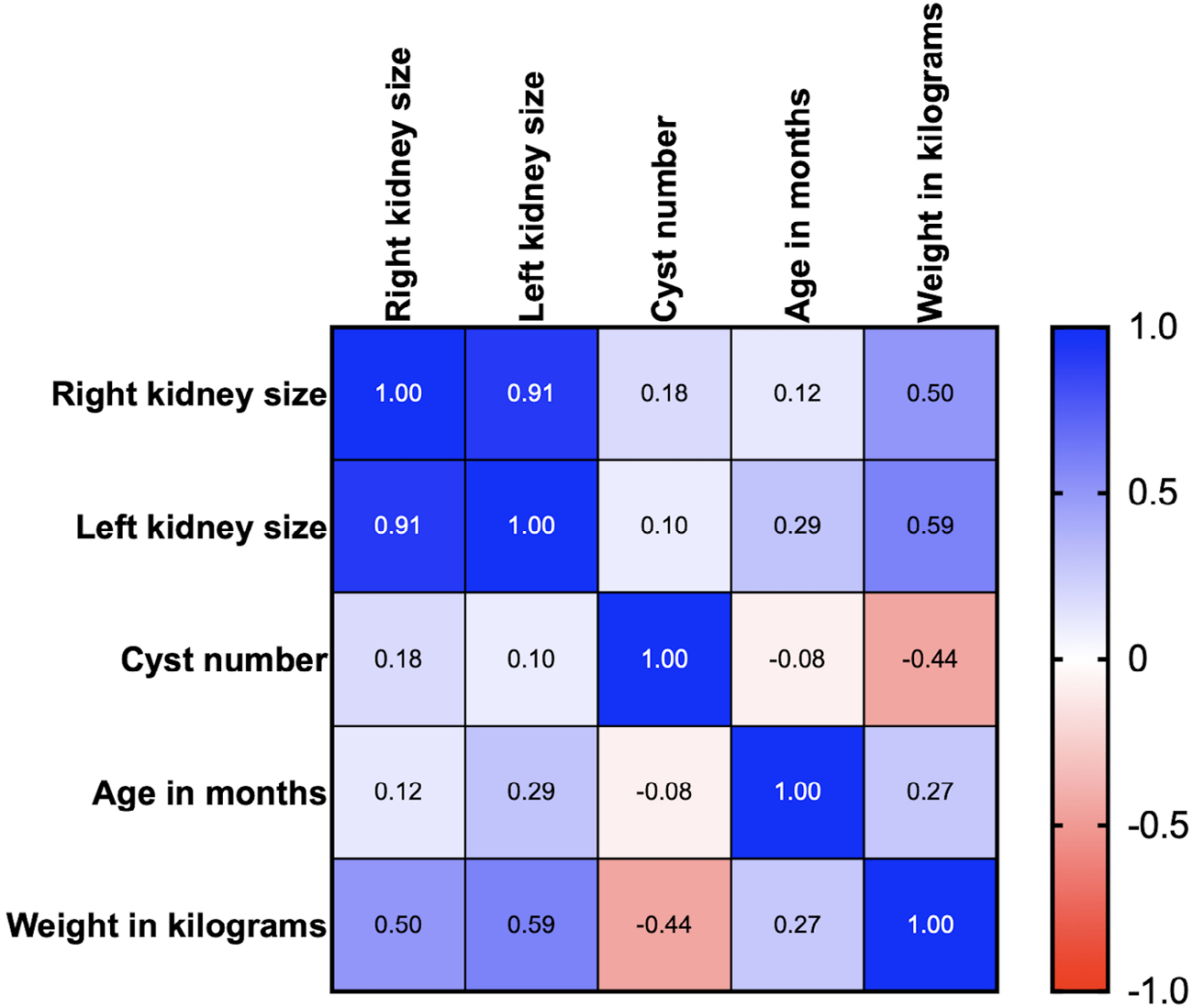
Correlation between Polycystin-1 (PKD1), ultrasonography profiles, and clinical data (age and body weight).

**Figure 5.**
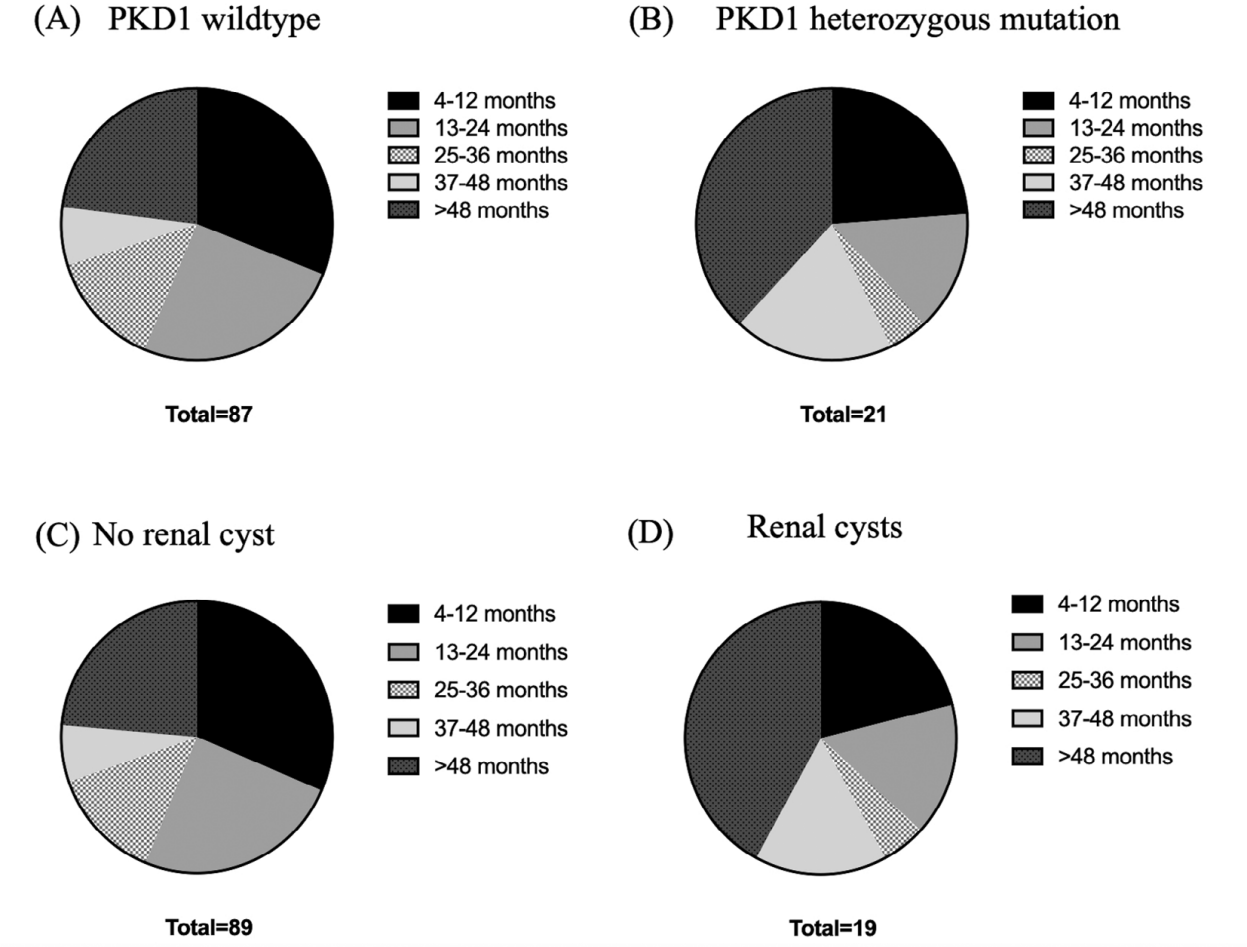
Relationships between ages, weight, and Polycystin-1 (PKD1) gene. (A) PKD1 wild-type, (B) PKD1 heterozygous mutation, (C) no renal cyst and (D) renal cysts.

Age, body weight, right kidney size, left kidney size, and the number of cysts were analyzed using correlation matrix analysis ( Figure 4). The results showed that age and weight were negatively related to the number of cysts. However, the weight was related to kidney size. There is likely no gene mutation if the cyst is not found at less than 36 months of age. Cysts are usually found in small cats weighing between 2 and 4 kg ( Figure 5).

## Discussion

This study’s differential ultrasonographic profiles between cats with PKD1 mutations and wild-type cats may provide complementary tools for detecting and evaluating polycystic kidney disease. The findings in our study are consistent with previously reported findings that the formation of the cysts is closely related to PKD1 gene mutations with high sensitivity and specificity. ^18^ As well as that, reports from Italy and Taiwan revealed that the sensitivity of ultrasonography was approximately 78.6 to 92.6%, and the specificity was 91 to 100%. ^18^^,^^20^ However, a previous study reported that pure-breed Maine Coon cats developed cystic formation in renal parenchyma without PKD1 gene mutation. ^21^ Therefore, the gold-standard technique of PKD1 gene mutation, PCR, and DNA sequencing is still recommended for confirming the genotype and further breeding selection consideration.

Of the total 108 cats examined by ultrasound, 19 were confirmed to have renal cysts, and in every cat with cysts found, a PKD1 mutation was present. Previous studies have indicated age-related cyst numbers for an ultrasound to diagnose PKD1 mutations. The cysts detected are sufficient for a positive diagnosis in young cats up to 12 months of age. In addition, these criteria will help to determine a positive result for PKD1 mutations if multiple bilateral cysts are detected (>10 per kidney) or if cysts are not detected in older cats. The results from this study are similar to a previous study that reported a positive correlation between the detection of renal cysts and age, as cyst detection was increased in older animals. ^22^

The apoptosis pathway is associated with inflammation in PKD in humans and mouse models. ^23^^–^^26^ Bcl-2 and Erb-b2 may be related to the pathway of PKD in cats with sarcomeric protein mutations. Inhibition of the apoptosis pathway might be associated with renal fibrosis and cyst expansion in cats with PKD. ^27^^,^^28^

Caspases are another essential target in the apoptosis mechanism. ^29^^,^^30^ Caspases are involved in preserving kidney function, suggesting caspases as a potential new target for the treatment of PKD. ^25^

PKD1 mutation is caused by disrupting the mechanism that controls the tubular diameter. The cyst characteristics were reported to be involved in cAMP signal transduction. The vasopressin that acts on the V2 receptor is the most potent catalyst for cAMP formation. Vasopressin 2 receptors are located in the collecting ducts, connecting ducts, and thickened limbs of Henle, which is the area of cyst formation. Tolvaptan has been used in treating PKD because the anti-vasopressin 2 receptor plays an important role in cyst growth. ^31^^–^^33^ Other medications, such as metformin, which targets AMPK and cAMP signaling, have shown great promise in reducing cyst formation and cellular proliferation. ^34^^,^^35^ However, reports linking these novel therapeutic drugs with PKD in cats still require further confirmation. Future clinical trials of potential signaling pathways are crucial in PKD diagnosis and treatment in feline patients.

## Conclusions

The frequency of PKD1 mutations was high in Persian and Persian-related breed cats. Such modifications were associated with the polycystic kidney phenotype. Ultrasonographic examination of cats will be a helpful tool for the routine identification of carriers of the mutated gene for polycystic kidney disease in cats. The results from this study may provide important information on clinical presentation and a gene associated with feline PKD. Genetic insights from genes may enable a more precise diagnosis of the type of renal cyst, which allows treatment or prevention strategies. Furthermore, knowing a genetic susceptibility to kidney cysts early may help to more accurately predict those most at risk of chronic kidney disease in the future.

## Ethical approval

The work described in this manuscript involved using non-experimental (owned) animals. Established internationally recognized high standards (‘best practice’) of veterinary clinical care for the individual patient always followed ethical approval, and written informed consent for publication of the participants was obtained from the owners.

## Data Availability

Figshare: Raw data PKD1 in cats. https://doi.org/10.6084/m9.figshare.22957526.v1. ^36^ Figshare: Figures. https://doi.org/10.6084/m9.figshare.22957715.v1. ^37^ Figshare: consent form. https://doi.org/10.6084/m9.figshare.22958243.v1. ^38^ Repository: ARRIVE checklist for ‘PKD1 gene mutation and ultrasonographic characterizations in cats with renal cysts’. https://doi.org/10.6084/m9.figshare.23123414.v1. ^39^ Data are available under the terms of the Creative Commons Attribution 4.0 International license (CC-BY 4.0).
