# Supplementary material for: PKD1 gene mutation and ultrasonographic characterization in cats with renal cysts
Source: F1000Res. 2024 Feb 23;12:760. Originally published 2023 Jun 28. [Version 3] doi: 10.12688/f1000research.134906.3 (PMC11301141; doi:10.12688/f1000research.134906.3)
Supplement: Peer review history

# Reviewer report: Reviewer response for version 3

**DOI:** 10.5256/f1000research.162718.r306588

**Published:** 2024-08-05

**Authors:** Jin He

**Affiliations:** 1 Zhejiang University, Hangzhou, Zhejiang, China; 2 College of Animal Sciences, Zhejiang University, Hangzhou, Zhejiang, China

The manuscript by Jaturanratsamee et al. presents a comprehensive study investigating the correlation between polycystic kidney disease (PKD) and the PKD1 gene mutation in cats. Utilizing a cohort of 108 cats across various breeds, the authors employ a multimodal approach, including blood examination, biochemical tests, renal ultrasound, and genetic analysis via polymerase chain reaction (PCR) and DNA sequencing. The primary objective is to assess the prevalence of PKD1 gene mutations and to characterize the associated renal phenotypes using ultrasonography, with the ultimate goal of informing treatment strategies and prevalence estimates for feline PKD.

 The study's design is robust, incorporating a broad sample of cat breeds and a systematic methodology for data collection and analysis. The use of both imaging and genetic techniques provides a dual perspective on the disease's presentation, offering valuable insights into the phenotypic expression of the PKD1 mutation in cats. The manuscript also discusses the implications of these findings for clinical practice, emphasizing the potential of ultrasonography as a diagnostic tool for identifying PKD1 gene carriers.

 However, as with any research, there are aspects of the study that warrant further consideration and clarification. The following comments will address specific areas where additional information, refinement, or alternative approaches may enhance the study's validity, reproducibility, and overall contribution to the field of veterinary genetics and nephrology.

 1) Similar to Reviewer 1, I am curious about the selection of cats involved in the study. Are they typically normal-conditioned cats or sick ones? If they belong to the latter category, then the calculated ratios might be overestimated.

 2) The authors stated in the statistics section that paired t-tests were utilized in the study. However, I am unsure how a paired test could be applied to analyze Table 1. As a biologist with extensive experience in statistics teaching, I would argue that only the comparison between the left kidney size and the right kidney size could be analyzed using this method. Besides, normalization of kidney size with their respective body weights may be a better way of presenting the data.

 3) The authors mentioned the use of multiple linear models; however, I was unable to locate them in the manuscript.

 4) In Table 2, I am puzzled by how the overall creatinine level can be calculated as 2.21 when the constituent parts of the population are recorded as 1.95 and 2.00.

 5) The authors should include a discussion on SDMA in comparison to creatinine, which could benefit biologists with limited clinical backgrounds, since creatinine levels are commonly utilized in research.

 6) I am curious about the calculation of the odds ratio given that there are no false-negative cases. Additionally, the specificity presented differs from my own calculations.

 7) The PCR genotyping method may only cover a portion of the feline PKD1 gene, implying that the 'wild-type' (WT) cats might also harbor PKD1 mutations. This concern was raised by other reviewers, but it seems to me that the authors have not adequately addressed it.

 8) Is there a gold standard or diagnostic criterion in veterinary medicine for ADPKD, similar to the Ravine criterion used in human studies? Cyst formation could also be a result of other diseases.

 9) Considering the above comments, the authors should carefully select appropriate terminology to describe and discuss their findings. Their conclusions may only be valid within the context of the specific population studied, potential undetected PKD1 mutations, and the diagnostic methods employed.

Is the work clearly and accurately presented and does it cite the current literature?

Yes

If applicable, is the statistical analysis and its interpretation appropriate?

No

Are all the source data underlying the results available to ensure full reproducibility?

Partly

Is the study design appropriate and is the work technically sound?

Partly

Are the conclusions drawn adequately supported by the results?

Yes

Are sufficient details of methods and analysis provided to allow replication by others?

Partly

Reviewer Expertise:

Animal models of ADPKD. Statistical genetics and genomics.

I confirm that I have read this submission and believe that I have an appropriate level of expertise to confirm that it is of an acceptable scientific standard, however I have significant reservations, as outlined above.

## References

[1] : Ultrasonographic kidney length-to-abdominal aortic diameter for the diagnosis of feline chronic kidney disease: A preliminary study. *Vet World* .2023;16(5) : 10.14202/vetworld.2023.1114-1121 1114-1121 10.14202/vetworld.2023.1114-1121 37576749 PMC10420722

# Reviewer report: Reviewer response for version 2

**DOI:** 10.5256/f1000research.157775.r222872

**Published:** 2024-02-05

**Authors:** Stephen C Parnell

**Affiliations:** 1 University of Kansas Medical Center, Kansas, USA

In the manuscript “PKD1 gene mutation and ultrasonographic characterization in cats with renal cyst” authors Jaturanratsamee and others use ultrasonography to investigate the association between clinical presentation of polycystic kidney disease (PKD) in a population of non-experimental house cats including animals with or without feline PKD1 gene mutation. The stated purpose of the study is to guide treatment options and to help estimate the prevalence of PKD in cats.

 Animals enrolled in the study were examined by routine medical exam, ultrasonography, serum biochemistry, and targeted PCR analysis of the feline PKD1 gene. Relationships between genotype and phenotype are presented.

 The stated conclusions for the study are that the frequency of PKD1 mutations are high in Persian and Persian-related breeds, that PKD1 mutation correlates with the PKD phenotype, and that ultrasonography can be a helpful tool to identify carriers of the mutated feline PKD1 gene.

 The study is well-conducted for the most part, but I have a few questions and minor corrections that the authors should address.

 The items listed below should be addressed in a revised version.

1. This portion of the manuscript is not clear to me: *Of the total 108 cats examined by ultrasound, no cyst was found in 87 cats of the wild-type group. However, 19 cats were confirmed to have renal cysts, and in every cat with cysts found, a PKD1 mutation was present.*
  An older domestic shorthair with cysts but no evidence of the relevant PKD1 mutation was reported elsewhere in the manuscript and should be addressed again at this point.
  Is it possible that the 51-month specimen represents a novel PKD1 mutation? Were the cysts in this animal unilateral or bilateral?
2. Have additional PKD1 mutations been identified within cats, or is the c.10063 CA transversion the only mutation identified to date? If so then this is interesting given the very large number of unique PKD1 mutations in the ADPKD patient population, but raises a more fundamental question. The authors conclude that “ultrasonographic examination of cats will be a helpful tool for the routine identification of carriers of the mutated gene for polycystic kidney disease in cats.” The manuscript reports sensitivity of only 75% at 16 week of age - isn’t this close to sexual maturity? If there is a single founder mutation for PKD in cats then wouldn’t a PCR-based assay (which could be performed well before sexual maturity) be much more useful for diagnosis than ultransonography? The authors should discuss this, and also note the age of sexual maturity in cats.
3. The cats with mutant PKD1 are on average almost 17-months older than the wild-type cats. This seems non-random. How were study animals selected, and has there been any consideration of whether there could be selection biases that affect interpretation of the data?

The items listed below should be corrected or amended in the final version.

1. In Figure 3 B/C are both labelled as Right Kidney. One should be the data for the left kidney.
2. In Figure 4 the chemical name is listed as “alanine” but it should be “adenine”. Please indicate the resulting protein mutation caused by this transversion.
3. In Table 2 please indicate the percentage of cystic cats in which the cysts are unilateral vs bilateral.

Is the work clearly and accurately presented and does it cite the current literature?

Yes

If applicable, is the statistical analysis and its interpretation appropriate?

Yes

Are all the source data underlying the results available to ensure full reproducibility?

Yes

Is the study design appropriate and is the work technically sound?

Yes

Are the conclusions drawn adequately supported by the results?

Partly

Are sufficient details of methods and analysis provided to allow replication by others?

Yes

Reviewer Expertise:

NA

I confirm that I have read this submission and believe that I have an appropriate level of expertise to confirm that it is of an acceptable scientific standard, however I have significant reservations, as outlined above.

# Response

**Published:** 2024-02-15

**Authors:** Soontaree Petchdee

**Affiliations:** Veterinary Clinical Science, Faculty of Veterinary Medicine, Kasetsart University, Kamphaengsaen, Thailand

We have provided a point-by-point reply to each point raised by the reviewers as a separate file and indicated any changes or corrections within the text in blue on the resubmitted manuscript. Please see below for the list of changes.

**Comments to the Author;**

**Reviewer** **2:**

**This portion of the manuscript is not clear to me: Of the total 108 cats examined by ultrasound, no cyst was found in 87 cats of the wild-type group. However, 19 cats were confirmed to have renal cysts, and in every cat with cysts found, a PKD1 mutation was present.** **An older domestic shorthair with cysts but no evidence of the relevant PKD1 mutation was reported elsewhere in the manuscript and should be addressed again at this point.** **Is it possible that the 51-month specimen represents a novel PKD1 mutation? Were the cysts in this animal unilateral or bilateral?**

**Answer to** **the reviewer:** We apologize for the unclear on this point. We have edited this point in the revised manuscript in the discussion section.

 The 51-month-old cat may represent a PKD1 mutation, and it usually correlates with a bilateral cyst. The cysts detected are sufficient for a positive diagnosis in young cats up to 12 months of age. In addition, these criteria will help to determine a positive result for *PKD1* mutations if multiple bilateral cysts are detected (>10 per kidney) or if cysts are not detected in older cats. The results from this study are similar to a previous study that reported a positive correlation between the detection of renal cysts and age, as cyst detection was increased in older animals.

**Have additional PKD1 mutations been identified within cats, or is the c.10063 CA transversion the only mutation identified to date? If so then this is interesting given the very large number of unique PKD1 mutations in the ADPKD patient population, but raises a more fundamental question. The authors conclude that “ultrasonographic examination of cats will be a helpful tool for the routine identification of carriers of the mutated gene for polycystic kidney disease in cats.” The manuscript reports** **a sensitivity of only 75% at 16 week** **s of age - isn’t this close to sexual maturity? If there is a single founder mutation for PKD in cats then wouldn’t a PCR-based assay (which could be performed well before sexual maturity) be much more useful for diagnosis than ultrasonography? The authors should discuss this and also note the age of sexual maturity in cats.**

**Answer to** **the reviewer:** We apologize for the unclear on this point. We have edited this point in the revised manuscript in the discussion and conclusion section.

 Similar to our study, in the previous study, ultrasound provided more sensitivity when diagnosing cats of maturity age, which had a sensitivity of 91% when performed at 36 weeks of age. In our study, we suggested that ultrasonographic examination of cats will be a helpful tool for the routine identification of carriers of the mutated gene for polycystic kidney disease in mature cats.

 A previous study reported sexual maturation in the domestic cat based on histological examination of seminiferous tubules, cats were classified as juvenile (<9 months; only spermatogonia and Sertoli cells present), pubertal (≥9 months to <1 year; complete spermatogenesis), and adult (>2 years; spermiogenesis complete and cauda epididymis filled with spermatozoa).

 (Ref.) Schön, J., Neumann, S., Wildt, D. E., Pukazhenthi, B. S., & Jewgenow, K. (2009). Localization of oestrogen receptors in the epididymis during sexual maturation of the domestic cat. *Reproduction in domestic animals = Zuchthygiene*, *44 Suppl 2*, 294–301. https://doi.org/10.1111/j.1439-0531.2009.01391.x

**The cats with mutant PKD1 are on average almost 17** **months older than the wild-type cats. This seems non-random. How were study animals selected, and has there been any consideration of whether there could be selection biases that affect** **the interpretation of the data?**

**Answer to** **the reviewer:** We appreciate the helpful comment and apologize for the unclear on this point. The animals in this present study were truly randomized. We were random and blind in all the processes. The person who performed the ultrasounds, the person who recorded the genetic parameters, and the person who analyzed the data were different persons.

**In Figure 3 B/C are both labelled as Right Kidney. One should be the data for the left kidney.**

**Answer to** **the reviewer:** We apologize for this point. We have edited this point in the revised manuscript

**In Figure 4 the chemical name is listed as “alanine” but it should be “adenine”. Please indicate the resulting protein mutation caused by this transversion.**

**Answer to** **the reviewer:** We apologize for this point. We have edited this point in the revised manuscript

**In Table 2 please indicate the percentage of cystic cats in which the cysts are unilateral vs bilateral.**

**Answer to** **the reviewer:** We apologize for this point. We have edited this point in the revised manuscript

 Best regards

 Soontaree Petchdee

 Corresponding author

# Reviewer report: Reviewer response for version 2

**DOI:** 10.5256/f1000research.157775.r218780

**Published:** 2023-11-03

**Authors:** Leslie A. Lyons

**Affiliations:** 1 Department of Veterinary Medicine and Surgery, College of Veterinary Medicine, University of Missouri, Columbia, MO, USA

Authors have made appropriate corrections.

Is the work clearly and accurately presented and does it cite the current literature?

Yes

If applicable, is the statistical analysis and its interpretation appropriate?

Yes

Are all the source data underlying the results available to ensure full reproducibility?

Partly

Is the study design appropriate and is the work technically sound?

Yes

Are the conclusions drawn adequately supported by the results?

Partly

Are sufficient details of methods and analysis provided to allow replication by others?

Yes

Reviewer Expertise:

Genetics PKD feline diseases

I confirm that I have read this submission and believe that I have an appropriate level of expertise to confirm that it is of an acceptable scientific standard.

# Reviewer report: Reviewer response for version 1

**DOI:** 10.5256/f1000research.147998.r183547

**Published:** 2023-10-16

**Authors:** Leslie A. Lyons

**Affiliations:** 1 Department of Veterinary Medicine and Surgery, College of Veterinary Medicine, University of Missouri, Columbia, MO, USA

The manuscript by Jaturanratsamee et al., entitled “ **PKD1 gene mutation and ultrasonographic characterization in cats with renal cysts” **describes the evaluation of 108 cats of variable breeds. Blood examination and biochemical tests were evaluated. For cyst detection, renal ultrasound was performed. The PKD1 gene mutation was identified *via *polymerase chain reaction (PCR) and DNA sequencing. 19.44% (21 of 108) of cats had PKD1 mutations, a high prevalence in Persian and Persian-related breed cats.

 The authors present a dataset including 108 cats in which 21 had the variant for PKD1. The dataset is important however the authors need to do some different presentations that would be of more significant value to the veterinary and PKD research communities. Hence. “partly” for the interpretation of data.  Partly for data presentation as all US images are not available.

 The authors should note 87 cats had no cysts at all. What is the frequency of singular cysts in humans, I think this supports cats rarely have singular cysts, or at least less commonly than humans.

 18 Persian cats, 49 Persian-related breed cats, 22 domestic shorthair cats, and 19 Bengal cats – need table with age at US for each cat – the figures are less useful. Produce a summary table that summarizes groups, per breed, the cats without the PKD1 variant and then provides each breed cat that had PKD individually.  Which cat had PKD1 variant but no cysts – what was the cat’s age?  Mention this in the text.

 Scottish Folds are popular in eastern countries, however, the rate of PKD is high – contrast to other, recent population data suggesting PKD1 is a very low rate in Persians and other breeds.

 BUN high for both populations – age correlation is important – please comment.

 PKD is one of the most common hereditary disease – actually simple Mendelian diseases in cats.  HCM may be more common but has a complex inheritance.

 Was the hair clipped for the US evaluations or alcohol or gel applied for US?

 Overall creatinine for overall population was 2.21 _ 0.19 – odd not intermediate – correct?

 Which was most predictive of PKD – renal failure, number of cysts, age, BUN, SDMA?   Any cats in renal failure?

 Was left to right kidney size significantly different in PKD1 cats?

 Figure 4 would be better with biochemistries and cysts, kidney size, in affected cats versus normal.  More figures like this, less pie charts.

 The results showed that age and weight were negatively related to the number of cysts?  I think this is misstated in results.

 "The feline Polycystin-1 (PKD1) gene was examined for the point of 85% of causal mutations" – not sure what this means.

Is the work clearly and accurately presented and does it cite the current literature?

Yes

If applicable, is the statistical analysis and its interpretation appropriate?

Yes

Are all the source data underlying the results available to ensure full reproducibility?

Partly

Is the study design appropriate and is the work technically sound?

Yes

Are the conclusions drawn adequately supported by the results?

Partly

Are sufficient details of methods and analysis provided to allow replication by others?

Yes

Reviewer Expertise:

Genetics PKD feline diseases

I confirm that I have read this submission and believe that I have an appropriate level of expertise to state that I do not consider it to be of an acceptable scientific standard, for reasons outlined above.

# Response

**Published:** 2023-10-18

**Authors:** Soontaree Petchdee

**Affiliations:** Veterinary Clinical Science, Faculty of Veterinary Medicine, Kasetsart University, Kamphaengsaen, Thailand

**Reviewer1:**

**The authors present a dataset including 108 cats in which 21 had the variant for PKD1. The dataset is important however the authors need to do some different presentations that would be of more significant value to the veterinary and PKD research communities. Hence. “partly” for the interpretation of data.  Partly for data presentation as all US images are not available.**

**The authors should note 87 cats had no cysts at all. What is the frequency of singular cysts in humans, I think this supports cats rarely have singular cysts, or at least less commonly than humans.**

**Answer to** **the reviewer:** We apologize for this point. We have edited this point in the revised manuscript in the discussion section.

 Of the total 108 cats examined by ultrasound, no cyst was found in 87 cats of the wild-type group. However,19 cats were confirmed to have renal cysts, and in every cat with cysts found, a *PKD1* mutation was presented.

**18 Persian cats, 49 Persian-related breed cats, 22 domestic shorthair cats, and 19 Bengal cats – need** **a table with age at US for each cat – the figures are less useful. Produce a summary table that summarizes groups, per breed, the cats without the PKD1 variant and then provide each breed cat that had PKD individually.**

**Which cat had PKD1 variant but no cysts – what was the cat’s age?  Mention this in the text.**

**Answer to** **the reviewer:** We appreciate the helpful comment. We have edited this point in the revised manuscript in Table 3 and mentioned more information in the results part. We apologize for this point.

 However, there was a 51-month-old domestic shorthair cat without the *PKD1* gene mutation developing the cysts in renal parenchyma. Table 3 demonstrates the number of cats in various breeds with *PKD1* gene mutation and renal cyst development.

**Scottish Folds are popular in eastern countries, however, the rate of PKD is high – contrast to other, recent population data suggesting PKD1 is a very low rate in Persians and other breeds.**

**Answer to** **the reviewer:** We appreciate the helpful comment. We have edited this point in the revised manuscript in the discussion section.

 This study found that the rate of *PKD1* gene mutation was high in Persian and Persian-related breed cats including Scottish Folds cats, Previous reports has been suggested that *PKD1* mutation was found at very low rates in Persians and other breeds which is contrary to our results. The high number of *PKD1* mutations that had been reported may be due to the Persian and Persian-related breed cats being a popular cat breed in eastern countries and Thailand.

**BUN** **is high for both populations – age correlation is important – please comment.**

**Answer to** **the reviewer:** We apologize for this point. We have edited this point in the revised manuscript in the results and discussion part.

 As indicated in Table 2, the levels of BUN were mildly elevated than the normal reference value. However, Pearson’s correlation results revealed that there was no correlation between age and BUN levels in WT (r=0.1436 *P* = 0.62) and *PKD1* mutation group (r=0.3234 *P* = 0.099).

**PKD is one of the most common hereditary diseases – actually simple Mendelian diseases in cats.  HCM may be more common but has a complex inheritance.**

**Answer to** **the reviewer:** We apologize for this point. We have edited this point in the revised manuscript in the introduction part.

 Feline polycystic kidney disease (PKD) is an inherited disorder that develops cysts in the renal parenchyma.

**Was the hair clipped for the US evaluations or alcohol or gel applied for US?**

**Answer to** **the reviewer:** We apologize for this point. We have edited this point in the revised manuscript in the materials and methods part.

 In our ultrasonographic examination, all cats were clipped the hair in the abdominal area and the ultrasonographic gel was used in all cats.

**Overall creatinine for overall population was 2.21 _ 0.19 – odd not intermediate – correct?**

**Answer to** **the reviewer:** We apologize for this point. We have edited this point in the revised manuscript in the results part.

 Additionally, the results of average creatinine levels in overall cats were elevated. Nonetheless, our results demonstrated that increasing creatinine exceeding 2.0 mg% may slightly increase the possibility for renal cyst formation by 1.41 times (95% CI, 0.3827 to 5.1764, *P* = 0.6070).

**Which was most predictive of PKD – renal failure, number of cysts, age, BUN, SDMA?   Any cats in renal failure?**

**Answer to** **the reviewer:** We apologize for this point. We have edited this point in the revised manuscript in the results part. The enrolled cats in this study are represented with either renal failure or non-renal failure.

 Therefore, cats with increasing SDMA, an age exceeding 36 months, body weight between 2-4 kg, and renal cyst formation from ultrasonographic examination are a high possibility of PKD1 gene mutation with PKD disorder.

 According to the elevated BUN in our study, the enrolled cats in this study are represented with either renal failure or non-renal failure. The high levels of BUN and increasing age were not related in contrast with the prior study from humans at that age and BUN indicated a positive correlation.

**Was left to right kidney size significantly different in PKD1 cats?**

**Answer to** **the reviewer:** We apologize for this point. We have edited this point in the revised manuscript in the results part.

 However, there was no statistically significant difference between right and left kidney size in cats affected with PKD1 mutation ( *P*=0.9980).

**Figure 4 would be better with biochemistries and cysts, kidney size, in affected cats versus normal. More figures like this, less pie charts.**

**Answer to** **the reviewer:** We apologize for this point. We have edited this point in the revised manuscript and added Figure 4.

**The results showed that age and weight were negatively related to the number of cysts?  I think this is misstated in results.**

**Answer to** **the reviewer:** We apologize for this point. We have edited this point in the revised manuscript in the discussion part. As in Figure 4, the results of matrix correlation show a negative correlation between the number of cysts and body weight (r=0.08) and age (r=0.44).

 Nevertheless, our study indicated a negative correlation between the age and the number of cysts in recruited cats. Moreover, the cats in this study have a negative correlation between cyst number and body weight. This could potentially result from the manifestation of PKD symptoms in cats with *PKD1* mutation.

**"The feline Polycystin-1 (PKD1) gene was examined for the point of 85% of causal mutations" – not sure what this means.**

**Answer to** **the reviewer:** We apologize for this point. We have edited this point in the revised manuscript in the introduction part.

 The polycystin-1 ( *PKD1*) gene mutation was indicated for 85% of human autosomal dominant polycystic kidney disease (ADPKD). The mutation of the *PKD1 *gene in cats carried heterozygous CA transversion at c.10063 in exon 29, resulting in impaired renal function.

 Best regards

 Soontaree Petchdee

 Corresponding author
